# Osteoarthritis and Total Joint Arthroplasty in Housing-Insecure Patients at a Safety Net Hospital in a Major Urban City

**DOI:** 10.1016/j.artd.2025.101773

**Published:** 2025-07-12

**Authors:** Abbott Gifford, Kelechi Nwachuku, Lisa Bonsignore-Opp, Paul Toogood, Derek Ward

**Affiliations:** a University of California-San Francisco School of Medicine, San Francisco, CA, USA; b University of California-San Francisco Department of Orthopaedic Surgery, San Francisco, CA, USA; c Orthopaedic Trauma Institute, San Francisco General Hospital, University of California, San Francisco, San Francisco, CA, USA

**Keywords:** Arthroplasty, Osteoarthritis, Homelessness, Social determinants of health, Safety net hospital

## Abstract

**Background:**

Osteoarthritis is the leading cause of disability among older adults in the United States. People experiencing homelessness (PEH) face worse health outcomes and higher rates of musculoskeletal diseases than housed individuals. Despite this burden, PEH often lack access to orthopaedic care. This project examines surgical outcomes among PEH and investigates where in the care process barriers to access may exist.

**Methods:**

New patient visits to the Zuckerberg San Francisco General Hospital Arthroplasty Clinic in 2022 were examined to establish a retrospective cohort. Patients were grouped by housing status, and data on demographics, disease severity, and comorbidities were collected. Analysis was performed using descriptive statistics and logistic regression. Presentation rate was calculated among clinic-presenting PEH and compared to a similarly captured population of patients on the San Francisco Health Plan.

**Results:**

Of 250 patients, 4 were unhoused and 41 were unstably housed. PEH and housing insecurity had worse Kellgren–Lawrence scores, higher rates of substance use, mental illness, HIV, and hepatitis C virus than stably housed patients. There were no differences in surgical progression, emergency department visits, readmission, reoperation, or follow-up. Significantly fewer PEH presented to clinic compared to those on the San Francisco Health Plan (X^2^ = 11.37, *P* = .0007).

**Conclusions:**

No differences in progression to surgery or surgical outcomes were found between housing groups. PEH accessed arthroplasty services less frequently than housed individuals. These findings suggest that PEH from the study population may be good surgical candidates and have limited access, but conclusions are limited by a short study follow-up.

## Introduction

Osteoarthritis (OA) affects more than 500 million individuals worldwide (7% of the global population) and is the leading cause of disability in older adults in the United States [1,2]. Modifiable risk factors for OA include physical activity and repetitive motion, obesity, metabolic disease, diet, and previous musculoskeletal injury [3]. A standard for progressive OA treatment is total joint arthroplasty (TJA), typically resulting in significant symptom improvement [4]. However, physicians often oppose performing this treatment on people experiencing homelessness (PEH) due to beliefs of higher postoperative complication rates and lack of attendance at follow-up appointments.

The United States Department of Housing and Urban Development defines homelessness as “an individual or family with a primary nighttime residence that is a public or private place not designed for or ordinarily used as a regular sleeping accommodation for human beings, including a car, park, abandoned building, bus or train station, airport, or camping ground;” this includes provisions for emergency shelters and transitional housing, motels, and hotels, staying with others, and at-risk homelessness [5]. In 2022, roughly 582,500 people were experiencing homelessness in the United States [6]. Generally, PEH and housing insecurity suffer from worse health outcomes than housed individuals [7]. A survey of PEH over the age of 50 in Boston found that 41.3% had difficulty walking, significantly higher than housed adults [8]. There is evidence to suggest that PEH are at higher risk for musculoskeletal injuries and diseases than the housed population, yet little work has been done to quantify the population-wide prevalence of diseases like OA [9,10]. Self-reported data estimate the prevalence of arthritis between 20%-45% in the unhoused population [9,11].

As of 2024, only 2 research articles on arthroplasty for PEH have been published. Neither study found significant contraindications to performing TJA in PEH; however, higher rates of emergency department (ED) presentation were noted in the PEH group. Researchers observed a higher rate of prosthetic joint infection among PEH readmitted to the hospital, though this result was not significant when controlled for confounders. In addition, 1 study demonstrated a 73% improvement in housing status among PEH following total knee arthroplasty [12,13].

There is limited literature which demonstrates that, though challenges exist in performing TJA among PEH, rates of revision, readmission, and major complications are comparable to the general population. However, anecdotal evidence suggests that these populations rarely receive TJA. There remains a significant need to better understand the demographics and clinical backgrounds of housing-insecure patients who receive arthroplasty care, as well as the outcomes of surgical intervention in this population. The primary aim of this study is to describe the population characteristics and postoperative outcomes of PEH and housing insecurity receiving care at a safety net hospital in an urban setting. As a secondary aim, the study explores potential points in the care provision process, such as clinic presentation, surgical optimization, and follow-up, where access barriers may exist.

## Material and methods

### Data source

All new patient visits at the Zuckerberg San Francisco General Hospital (ZSFG) Arthroplasty Clinic between January 1, 2022, and December 31, 2022, were identified. This hospital was selected as it has unique access to PEH due to the nature of San Francisco’s geography and medical care. This is the only safety net hospital in the city, and the city provides an insurance option at low or no cost to residents, making access to care at this hospital very inexpensive or free for patients without resources. Therefore, most patients in the group of interest likely seek care in this location. A retrospective chart review was conducted to extract patient demographics, descriptors of housing status, qualitative measures of disease status and comorbidities, and surgical characteristics. Further review identified patient insurance status and carrier to create control and study groups.

Patients above the age of 18 who presented to the Arthroplasty Clinic at ZSFG who, at the time of presentation, were homeless or experiencing housing insecurity were included in the study group. All patients who are scheduled for surgery through the arthroplasty clinic at ZSFG were asked, "Do you live in a house, apartment, SRO?" as part of standard clinic intake questionnaires. In this context, SRO stands for “Single Room Occupancy” and is a type of setting that meets the criteria for marginal housing. If these data were unavailable for certain patients, addresses were checked against known SROs and shelters. This information allowed us to create patient cohorts of those who were unhoused, marginally housed, and housed. Patients included in the marginally housed group were either living in an SRO, incarcerated, or documented as having other marginal housing at the time of clinic presentation. It was assumed that the majority of PEH in San Francisco seek care at ZSFG as the sole safety net hospital for the county. Likewise, patients on the San Francisco Health Plan (SFHP) almost exclusively receive care at this location and have a known percentage of PEH enrollees. These 2 captured populations and their relative presentation rates at the Arthroplasty Clinic at ZSFG were used to determine a difference in presentation based on housing status.

The 2022 San Francisco Homeless Count and Survey was used to determine the total number of PEH in San Francisco in 2022. The San Francisco Health Authority, San Francisco Health Plan 2022 Audit provided the count of all patients on the SFHP during the study window.

Due to the small sample size of unhoused patients, for further analysis beyond presentation rate, a composite measure (“Unstably Housed”) was made of patients who were homeless, marginally housed, and incarcerated. Kellgren–Lawrence (KL) scores were calculated for each patient based on the narrative provided in preoperative radiology reports. Grades 0-4 were given per qualitative interpretation, representing none, doubtful, minimal, moderate, and severe OA.

This study received approval from the University of California San Francisco Institutional Review Board and Research Protocol Approval from the ZSFG and the San Francisco Department of Public Health.

### Data analysis

Demographic and clinical traits of the research cohort were summarized with descriptive statistics. Fisher exact tests and Pearson's *Chi*-squared test with Yates' continuity correction were used to compare categorical and continuous variables between groups. Multivariate logistic regression was used to compare the surgical outcomes between the study and control groups. All statistical analyses were conducted using R version 4.1.3 (2022-03-10) within RStudio via University of California San Francisco Research Analysis Environment. Significance is defined as *P* < .05.

## Results

Among all patients seen in the ZSFG Arthroplasty Clinic between January 1, 2022, and December 31, 2022, we identified 250 patients who met the inclusion criteria. Four of the patients included in the analysis were unhoused, 41 were unstably housed, 1 was incarcerated, and 204 were stably housed.

Patients in the unstably housed group were more likely to be male (80.4% vs 34.4%, *P* < .0001) and were significantly younger than the stably housed group (63.4 vs 60.3, *P* = .0186). The unstably housed group had an increased proportion of Black and White patients. In contrast, the stably housed cohort had a higher proportion of Asian and American Indian and Alaskan Native patients (*P* = .0031).

The unstably housed cohort had higher rates of active alcohol use (18.2% vs 3.9%, *P* = .0005), opioid use (22% vs 0.5%, *P* < .0001), stimulant use (30% vs 3%, *P* < .0001), and smoking (56% vs 18.6%, *P* < .0001). Patients in the unstably housed group had higher rates of mental illness (40% vs 22.2%, *P* = .022) and a worse KL score when presenting at clinic (3.6 vs 3.2, *P* = .0006). The unstably housed cohort had significantly higher rates of HIV infection (13% vs 2.9%, *P* = .011) and hepatitis C virus (HCV) (23.9% vs 5.9%, *P* = .0004). No other differences in patient demographics or comorbidities were significant (Table 1).

**Table 1:** Summary of demographics, comorbidities, and surgical outcomes for patients by housing status.

Demographics	Cohorts		
	Overall	Unstably housed	Stably housed
Mean Age	62.8	60.3	63.4
Sex			
Male	40.4	80.4	31.4
Female	59.6	19.6	68.6
Ethnicity (%)			
Hispanic/Latino	26.8	17.4	29.8
Not Hispanic or Latino	73.2	82.6	70.8
Race (%)			
White	29.6	41.3	27.2
Black	21.6	34.8	18.8
Asian	20.8	4.4	24.8
American Indian Alaskan Native	1.6	2.2	1.5
Other	25.6	17.4	27.7
Insurance Status (%)			
Insured	98.4	100%	98.5%
Comorbidities			
Alcohol Use	7.6%	18.2%	3.9%
Opioid Use	5.2%	22.0%	0.5%
Stimulant Use	11.6%	30.0%	3.0%
Smoking	35.6%	56.0%	18.6%
Mental Illness	25.2%	40.0%	22.2%
HIV	4.8%	13.0%	2.9%
HCV	9.2%	23.9%	5.9%
Average KL Score	3.24	3.62	3.17
Outcomes (%)			
Surgery	21.6	23.9	22.4
Injections	28.4	37.0	26.7
Follow-Up Attendance	33.3	36.63	25.6
90-d ED Visits	11.1	18.2	16.2
90-d Readmission	5.6	9.1	4.7
1-y Revision Surgeries	1.8	0.0	2.3

There was no difference in progression to surgery or in-clinic injections between groups (*P* > .05). While a higher KL score at presentation was associated with future surgery, when controlled for KL score in the multivariate analysis, there was still no significant difference between groups on progression to surgery (*P* > .05). For patients who underwent surgery, there were no statistically significant differences between the stable housing group and the unstable housing group in attendance at follow-up visits, 90-day ED visits, 90-day readmissions, and 1-year revision surgeries (all *P* > .05) (Table 1).

In the calendar year 2022, there were an estimated 20,000 PEH in San Francisco, and 168,555 individuals on the SFHP. As a proportion of the respective populations, significantly fewer PEH (N = 4) presented to the ZSFG Arthroplasty Clinic than those on the SFHP (*N* = 166) (*X*^2^ = 11.4, *P* = .0007). Of the PEH who presented to clinic, 3 were enrolled in SFHP, indicating an overlap between the 2 groups. When the overlapping population was removed from the analysis, the difference between groups remained significant (*X*^2^ = 14.8, *P* = .0001). Directly comparing presentation rates shows that PEH were 5 times less likely to present at clinic compared to housed patients. Despite a small effect size (w = 0.009), the analysis was adequately powered (97%) to detect a statistically significant difference.

## Discussion

Compared to housed patients, there were no significant differences in the rate of surgery, attendance to follow-up visits, 90-day ED visits, 90-day readmissions, and 1-year revision surgeries in the unstably housed group. These results challenge the preconception of PEH as poor surgical candidates and are an indicator of successful medical and social management in the arthroplasty setting.

Previous research has demonstrated that PEH and housing insecurity attend scheduled medical appointments less consistently than housed individuals [13]. This is most often attributed to a lack of access (transportation, communication barriers, transient housing) and poor experiences with health-care provision [14]. However, our data indicate that previous findings may not be generalizable across all populations. In our cohort, PEH and housing insecurity had follow-up attendance rates similar to those of stably housed patients. These data challenge prevailing assumptions and highlight the potential impact of destigmatizing perceptions of vulnerable populations.

Furthermore, these data corroborate existing work on arthroplasty outcomes among PEH and housing insecurity, which found no differences between housed and unhoused patients in readmission and revision surgery [12,13]. Our results diverge from those of Niu et al., who found that PEH were 15 times more likely to present to the ED within 90 days of surgery. However, Niu et al.’s analysis was limited to PEH, whereas our cohort included both PEH and patients with broader indicators of housing instability [13]. This discrepancy may reflect meaningful differences in the composition and lived experiences of the study populations. Future research is warranted to further delineate outcomes between these distinct yet overlapping groups and to explore the contextual factors that shape postoperative trajectories.

Prior work suggests that PEH and housing insecurity face challenges in the presurgical period with the management of pre-existing conditions. They are more likely to have active HIV and HCV, Methicillin-resistant Staphylococcus aureus, and substance use disorder [[15], [16], [17], [18]]. Our findings support the existing data, as PEH and housing insecurity were more likely to have HIV and HCV, alcohol, opioid, stimulant use, and mental health comorbidities. PEH face more barriers than housed individuals with medication adherence and surgery-specific procedures such as showers and bowel prep [19,20]. In the current study, PEH and housing insecurity were just as likely as housed patients to undergo surgery once they presented to clinic. While differences in comorbidities were statistically significant, they did not impact medical optimization for surgery.

Our study suggests that PEH in San Francisco are accessing arthroplasty services at a rate lower than those who are housed. When the high rate of musculoskeletal injury and arthritis in the unhoused population is considered, this gap in access widens considerably. Abel et al 2022 use the phases of surgical care framework to understand the barriers facing homeless surgical patients [20]. The current study shows that the disparity in utilization of arthroplasty services begins before patients arrive at clinic, rather than in the preoperative period.

To date, no other studies have investigated the rate of clinic presentation for orthopaedic care among PEH. However, research on more general health-care utilization points to structural barriers that prevent PEH from obtaining health care, such as lack of health insurance, financial difficulties, competing priorities such as food and housing, and mistrust of the health-care system [21]. Though the current study found no differences between the unstably housed and stably housed cohorts on insurance status, many of the other factors affecting access to care among PEH are not routinely screened for. Further research on patient experience is important to understand the unhoused orthopaedic patient more deeply.

Significant demographic differences exist between the housed and unstably housed groups. The unstably housed group was more likely to be male and was younger than the housed cohort. There were also differences in the racial and ethnic makeup of these groups. These factors have been shown to independently impact outcomes in total joint surgery [[22], [23], [24]]. While the current study found no differences in outcomes between the study groups, these highlighted demographic differences may limit the interpretation of the results. In addition, several of the comorbidities found in the unstably housed group, including HIV and HCV infection, alcohol use, preoperative opioid use, and smoking, have been shown to be risk factors for worse outcomes after TJA [25,26]. Nui et al. found no difference in readmission and reoperation between homeless patients with high rates of alcohol use and opioid use and housed patients without such comorbidities [13]. However, the lack of significant differences in postoperative outcomes in the setting of adverse comorbidities could represent an insufficient follow-up period and inability to detect such outcomes in the current study.

Retrospective studies are subject to several sources of bias, including measurement, selection, and confounding bias. Though efforts were made to limit these biases through careful study design and multivariate regression, it is impossible to eliminate them. A particular limitation of the current study is the comparison of PEH and those on the SFHP. Both were believed to be populations completely captured at ZSFG as the safety net hospital for San Francisco County. However, it is possible that select patients were instead treated at other sites, such as the San Francisco Veterans Affairs hospital or other sites that accept private insurance. Reasonable next steps would be to perform a separate analysis of PEH outcomes using well-matched, housed controls. The current project faced significant challenges in determining a finite study population, as the counts of PEH from the 2022 San Francisco Homeless Count and Survey and the number of PEH on SFHP are well-informed estimates.

## Conclusions

There were no significant differences between PEH and housing insecurity and housed patients who received care at a safety net hospital’s arthroplasty clinic in postoperative ED utilization, surgical outcomes, and attendance at follow-up appointments. PEH presented to clinic at a disproportionately low rate when compared to housed patients and relative to their documented burden of OA and musculoskeletal injury. Our findings highlight that TJA may be a safe and effective option for PEH and housing insecurity, who face a high disease burden and barriers when accessing care. While results suggest that patients experiencing homelessness could achieve good outcomes under specific circumstances, the low sample size and follow-up duration require caution and further study. These results represent an important first step toward addressing disparities in musculoskeletal care and identifying opportunities to improve access and equity in surgical treatment for vulnerable populations.

## Conflicts of interest

Derek Ward is a consultant for DePuy and Smith & Nephew and owns Stock in Visie.

The other authors declare there are no conflicts of interest.

For full disclosure statements refer to https://doi.org/10.1016/j.artd.2025.101773.

## CRediT authorship contribution statement

**Abbott Gifford:** Writing – review & editing, Writing – original draft, Project administration, Methodology, Investigation, Formal analysis, Data curation, Conceptualization. **Kelechi Nwachuku:** Writing – review & editing, Methodology, Investigation. **Lisa Bonsignore-Opp:** Writing – review & editing. **Paul Toogood:** Writing – review & editing, Supervision. **Derek Ward:** Writing – review & editing, Supervision, Methodology, Conceptualization.

## Appendix A. Supplementary data

Conflict of Interest Statement for Bonsignore-Opp

Conflict of Interest Statement for Toogood

Conflict of Interest Statement for Gifford

Conflict of Interest Statement for Nwachuku

Conflict of Interest Statement for Ward
